# Risk Factors and Level of *Listeria monocytogenes* Contamination of Raw Pork in Retail Markets in China

**DOI:** 10.3389/fmicb.2018.01090

**Published:** 2018-05-29

**Authors:** Hua Li, Pengfei Wang, Ruiting Lan, Lijuan Luo, Xiaolong Cao, Yi Wang, Yan Wang, Hui Li, Lu Zhang, Shunshi Ji, Changyun Ye

**Affiliations:** ^1^State Key Laboratory of Infectious Disease Prevention and Control, Collaborative Innovation Center for Diagnosis and Treatment of Infectious Diseases, National Institute for Communicable Disease Control and Prevention, Chinese Center for Disease Control and Prevention, Beijing, China; ^2^Tongzhou District Center for Disease Control and Prevention, Beijing, China; ^3^School of Biotechnology and Biomolecular Sciences, University of New South Wales, Sydney, NSW, Australia; ^4^Beijing Changping Institute for Tuberculosis Prevention and Treatment, Beijing, China; ^5^Department of Microbiology, Guizhou Medical University, Guiyang, China

**Keywords:** *Listeria monocytogenes*, retail market, environment, insects, cross contamination

## Abstract

*Listeria monocytogenes* can contaminate various foods via food processing environments and contamination of raw materials. There is a limited understanding of *L. monocytogenes* transmission in retail market and the role of insects in *L. monocytogenes* transmission in the retail environments. To better understand the risk factors of raw pork contamination, the prevalence of *L. monocytogenes* was examined in raw pork, retail environments and insects in a retail market over a 6-month period from March to August in 2016 in Beijing, China. A total of 2,789 samples were collected, including 356 raw pork samples, 1,392 meat contact surface swabs (MCS), 712 non-meat contact surface swabs (NMCS) and 329 insect samples. Overall, 424 (15.20%) of the samples were found to be contaminated by *L. monocytogenes*. Analyzed by serotyping, multilocus sequence typing and pulsed-field gel electrophoresis, the 424 *L. monocytogenes* isolates were divided into three serotypes (1/2c, 1/2a and 3a), 15 pulsotypes (PTs) and nine sequence types (STs), 1/2c/PT4/ST9 (244/424, 58%) was the most prevalent type of *L. monocytogenes* strains. The raw pork, MCS of the environments and insects were contaminated with higher levels of *L. monocytogenes* than NMCS of the environments, which suggested that cross contamination of *L. monocytogenes* between raw pork and the environment existed in the retail market, and long-term contaminated surfaces and vector insects would act as high risk factors to transmit *L. monocytogenes* to raw pork. Thus more effective strategies are needed to reduce the risk of retail pork meat contamination by *L. monocytogenes* and prevent foodborne human listeriosis.

## Introduction

*Listeria monocytogenes* is a food-borne pathogen that causes severe infections in humans (Zhou et al., 2010; Galvao et al., 2012; Pagliano et al., 2017; Lowe et al., 2018), especially the elderly, pregnant women, newborns and immunocompromised individuals (Fleming et al., 1985; Gilmour et al., 2010; Lamont et al., 2011). The diseases caused by *L. monocytogenes* include meningitis, sepsis, gastroenteritis, neonatal infections and fetal loss (Lamont et al., 2011; Pagliano et al., 2017; Lowe et al., 2018). A total of 256 cases of listeriosis were reported from 1964 to 2013 in China, involved 48 perinatal women, 86 neonatal and 122 non-perinatal women and more sporadic cases of listeriosis were reported in recent years (Wu et al., 2008; Zhou et al., 2010; Sun et al., 2016). Listeriosis outbreaks have been reported in many countries including Japan, the United States and Europe (European Food Safety Authority, 2011; Miya et al., 2015; Self et al., 2016). Human infections caused by *L. monocytogenes* have become a global health concern.

*Listeria monocytogenes* is ubiquitous in the environment (Wagner and Stessl, 2014; Conficoni et al., 2016). It can adhere to many surfaces and form biofilms, and has the ability to survive and grow at low temperatures, a wide range of pH, high salt concentrations and low water activity (Duffy and Sheridan, 1997; Miettinen et al., 1999a,b; Freitag et al., 2009). *L. monocytogenes* has been isolated from various food products, including raw and cooked meats, milk products, seafood and vegetables (Ballesteros et al., 2011; Jami et al., 2014; Moosavy et al., 2014; Brown et al., 2016; Luo et al., 2017). High levels of *L. monocytogenes* contamination in raw pork in China have been reported (Zhang et al., 2007, 2013; Yang et al., 2008; Huo et al., 2015). In addition, *L. monocytogenes* can persist in food-processing environments causing recurrent contamination of final products (Nucera et al., 2010; O’Connor et al., 2010; Galvao et al., 2012). The bacterium enters the processing plant generally through raw materials, drainers, aerosols, personnel movements and food-processing facilities. In addition, a wide variety of ready-to-eat food, retail food and related environments have been found to be contaminated by *L. monocytogenes* in Canada, Italy, China and the United States in recent years (Hoelzer et al., 2011; Kovacevic et al., 2012; Simmons et al., 2014; Wu et al., 2015; Conficoni et al., 2016).

Currently, the understanding of risk factors of *L. monocytogenes* contamination of raw meat in retail market is limited, especially in China. A recent study showed that the retail environment played an important role in *L. monocytogenes* contamination in pork retail markets, but few environmental samples were collected (Luo et al., 2017). Insects such as flies and cockroaches can carry pathogens (Barreiro et al., 2013), but little is known about their role in *L. monocytogenes* transmission in China. Thus, we conducted this survey of *L. monocytogenes* contamination in raw pork, environments and insects in a raw pork retail market, and serotyping, multilocus sequence typing and pulsed-field gel electrophoresis were used to determine the characteristics of *L. monocytogenes* isolates and evaluate their potential risk to foodborne human listeriosis.

## Materials and Methods

### Sample Collection

Samples from the retail environments and raw pork were collected from 65 booths (which distributed in four rows) monthly for 6 months (March 2016–August 2016) in a raw pork retail market located in Beijing, China. The average temperature between March 2016 and August 2016 were 12, 20, 26, 30, 31, and 30°C respectively. A total of 2,789 samples were collected, including 356 raw pork samples, 2,104 raw pork retail environment swabs (1,392 MCS swabs from chopping boards and knives, the inner and outer surfaces of chest freezers, meat mincers, hands of persons, and 712 NMCS swabs from floors and walls), and 329 insects (185 flies and 144 cockroaches) samples. Environmental samples were collected using sterile swabs, rehydrated before use with 5 ml of sodium chloride solution (0.9) in sterile tubes. The sampling surface areas were 100–900 cm^2^ and samples were collected before cleaning and disinfection. Additionally, approximately 25 g of fresh raw pork sample contacting with chopping boards were collected in sterile bags. Most fresh raw pork in the retail market was sold within the day and any unsold meat were stored at -20°C in the freezer (each booth equipped one chest freezer for storage). Every 20 flies or cockroaches were pooled together to increase the sensitivity of isolation (Olsen and Hammack, 2000; Holt et al., 2007; Forster et al., 2009). All samples were maintained at 4°C for transport and storage, and were detected within 24 h.

### Isolation of *L. monocytogenes*

Isolation of *L. monocytogenes* was performed in accordance with ISO11290-1 (1996) method with partial modifications. Samples were examined with two steps of enrichment. Solid samples (pork pieces, 25 g) mixed with 225 ml Half Fraser broth and each environmental swab was mixed with 10 ml Half Fraser (Oxoid Ltd., Hampshire, United Kingdom) and were then incubated for 24 h at 30°C with shaking (220 rpm). Subsequently 1 ml of the above culture was transferred into 9 ml Fraser (Oxoid Ltd.) for 48 h at 37°C with shaking (220 rpm). Flies and cockroach samples were pulverized before culturing because that *L. monocytogenes* could exist on the body surface as well as the alimentary canal of a single fly (Pava-Ripoll et al., 2015). Enriched broth was then spread onto brilliance listeria agar (Oxoid Ltd.) for 24–36 h at 37°C. Presumptive *L. monocytogenes* colonies (3–5) were purified in brain heart infusion (BHI) agar (Landbridge Ltd., Beijing, China) for another 24 h at 37°C. For DNA extraction, pure strains from BHIA were transferred into 200 μl Tris-EDTA (TE) buffer, vibrating, blending and boiling the mixture at 100°C for 10–15 min, and then centrifuged for 3 min (13000 r/min), the supernatant was stored at -20°C. PCR amplification of *Listeria* genus and species of the isolates was determined by specific primers (Ryu et al., 2013).

### Serotyping of *L. monocytogenes* Isolates

Multiplex PCR serogrouping was carried out according to the method of Doumith et al. (2004). Subsequently, specific serotypes were determined by combining with the conventional Denka Seiken serotyping (Burall et al., 2011).

### Pulsed-Field Gel Electrophoresis (PFGE) Typing Based on *AscI* Digestion

Pulsed-field gel electrophoresis (PFGE) was performed according to the Pulse-Net standardized protocol for *L. monocytogenes* using *AscI* (Graves and Swaminathan, 2001; Sauders et al., 2003; Wang et al., 2012). *Salmonella enterica* serovar Braenderup strain H9812 restricted with *XbaI* was used for molecular weight determinations in all PFGE gels. The PFGE patterns were analyzed by using BioNumerics Software version 7.0 (Applied Maths, Saint-Martine, Belgium) and setting Dice coefficient and position tolerance at 1.0% for band comparison, and the pattern profiles were grouped together according to the unweighted pair group method with arithmetic average (UPGMA). The pulsotypes were standardized and compared with those in the database of PulseNet China.

### Multilocus Sequence Typing (MLST)

Multilocus sequence typing was performed based on sequence analysis of seven housekeeping genes *(abcZ, bglA, cat, dapE, dat, ldh, lhkA*). Lasergene’s Seqman software was used to compare the sequence. Analysis of allelic profiles or sequence types of the *L. monocytogenes* strain was done on the website^1^. Minimum spanning tree was created by BioNumerics Software version 7.0 (Applied Maths, Saint-Martine, Belgium).

### Statistical Analysis

To analyze the statistical significance (*p* < 0.05) of differences in the prevalence of *L. monocytogenes* in different booths, months and sample categories, chi square (X^2^) test and Fisher exact test were performed using SAS (version 9.4, SAS Institute Inc.).

## Results

### Prevalence of *L. monocytogenes* in Different Samples

A total of 2,789 samples were collected and 424 (15%) samples were positive for *L. monocytogenes*. *L. monocytogenes* isolation rate was 29, 16, 4, and 20% for raw pork, MCS, NMCS and insects samples respectively. The difference of positive rates among different sample types (raw pork, MCS, NMCS and insects) were significant (χ^2^= 130.972, *P* < 0.0001). Among MCS samples, contamination of chopping boards and knives (25%) was higher than that of outer surfaces of chest freezers (14%) and meat mincers (13%). The rates among 10 sample types in **Table 1** were statistically significant (χ^2^= 160.418, *P* < 0.01). The contamination rates of raw pork and MCS (excluding chopping board and knives, and meat mincers) varied significantly among different sampling months. The monthly contamination rate ranged from 14 to 22%, and incidences in May (22%), June (20%) and July (19%) were higher than the average rate of 15%, indicating that the months with higher contamination rate of *L. monocytogenes* was also the months with higher temperature (**Table 1**).

**Table 1:** Prevalence, temporal distribution, phenotypic and genotypic characteristics of *L. monocytogenes* isolates collected from raw pork, environments and insects in retail market.

Sample category	Sample details	No. of samples	No. (%) of isolates	Month variation		High-risk months	Serotypes (No. of isolates)	STs (No. of isolates)	PTs (No. of isolates)
				χ^2^	*P*				
Meat^a^	Raw pork	356	104 (29)	30	<0.0001	April, March, May	1/2c (75), 1/2a (15), 3a (14)	9 (75), 155 (14), 8 (11), 121 (2), 307 (2)	4 (62), 279 (11), 11 (11), 54 (11), 44 (2), 41 (2), 16 (2), 98 (1), 224 (1), 259 (1)
Meat contact surfaces (MCS^b^)	Chopping boards and knives	367	91 (25)	9.8	0.0796	April, July, May, June	1/2c (72), 1/2a (11), 3a (8)	9 (71), 155 (8), 8 (6), 121 (4), 204 (1), 307 (1)	4 (57), 279 (11), 11 (6), 16 (4), 44 (4), 9 (3), 54 (2), 98 (2), 41 (1), 5 (1)
	Inner surfaces of freezers	303	39 (13)	12.8	0.0254	July, August	1/2c (29), 1/2a (5), 3a (5)	9 (29), 155 (4), 8 (3), 121 (2), 705 (1)	4 (20), 279 (5), 54 (5), 9 (3), 11 (3), 16 (2), 270 (1)
	Outer surfaces of freezers	310	44 (14)	13.0	0.0236	July, May	1/2c (33), 3a (6), 1/2a (5)	9 (32), 155 (5), 121 (4), 8 (1), 204 (1), 307 (1)	4 (26), 16 (4), 279 (3), 54 (3), 98 (2), 9 (2), 5 (1), 11 (1), 44 (1), 73 (1)
	Meat mincers	100	13 (13)	—	0.0051^e^	June, May, April	1/2c (13)	9 (13)	4 (10), 7 (1)
	Hands	312	40 (13)	7.4	0.1946	July, May, March	1/2c (30), 1/2a (5), 3a (5)	9 (30), 155 (5), 121 (3), 8 (2)	4 (22), 9 (4), 16 (3), 279 (3), 11 (2), 44 (2), 54 (3), 224 (1)
Non-meat contact surfaces (MMCS^c^)	Floors	358	18 (5)	–	0.0294^f^	March, May	1/2c (16), 1/2a (2)	9 (16), 8 (1), 120 (1)	4 (12), 279 (4), 11 (2)
	Walls	354	10 (3)	–	0.1467^g^	May, July	1/2c (9), 3a (1)	9 (9), 155 (1)	4 (7), 279 (2), 44 (1)
Insects^d^	Flies	185	34 (18)	27.3	<0.0001	July, August	1/2a (16), 1/2c (15), 3a (3)	121 (16), 9 (15), 155 (3)	16 (16), 4 (15), 54 (2), 44 (1)
	Cockroaches	144	31 (22)	–	0.0004^h^	May	1/2c (16), 1/2a (5), 3a (10)	9 (15), 155 (10), 121 (5), 35 (1)	4 (14), 54 (7), 16 (5), 44 (3), 7 (1), 224 (1)

Total	–	2789	424 (15)	–	–	July, March, May	1/2c (308), 1/2a (64), 3a (52)	9 (305), 155 (50), 121 (36), 8 (24), 307 (4), 204 (2), 705 (1), 35 (1), 120 (1)	4 (245), 279 (41), 16 (36), 54 (33), 11 (25), 44 (14), 9 (12), 98 (5), 41 (3), 224 (3), 5 (2), 7 (2), 73 (1), 259 (1), 270 (1)

*^*a*^Meat, raw pork. ^*b*^MCS, meat contact surfaces. ^*c*^NMCS, non-meat contact surfaces. ^*d*^Insects, flies and cockroaches. ^*e,f,g,h*^Fisher exact test was performed.*

### Serotypes and Genotypic Characterizations of *L. monocytogenes* Isolates

**FIGURE 1:**
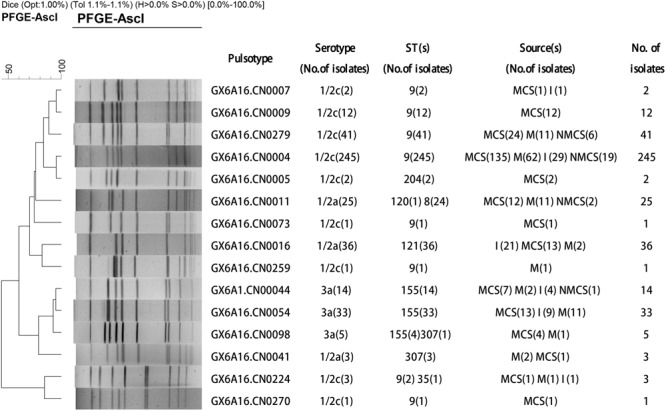
Dendrogram of pulsed-field gel electrophoresis (PFGE) patterns based on *AscI* digestion of 424 *L. monocytogenes* isolates from raw pork and environments in retail market. The PFGE patterns (PTs), serotypes, sequence types (STs), sources and number of strains are shown at right (MCS, meat contact surface; NMCS, non-meat contact surface; I, insects; M, raw pork).

**FIGURE 2:**
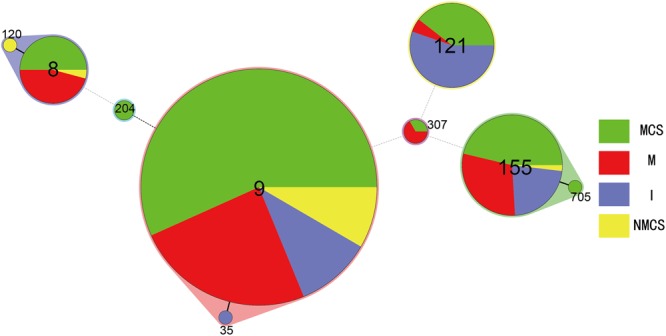
Minimum spanning tree analysis of *L. monocytogenes* isolates from raw pork, environments and insects in retail market located in China. The data analyzed was based on MLST result of the isolates presented in **Table 1**. Circles correspond to sequence types (STs), the size of each circle is proportional to the number of isolates in each ST. The minimum spanning tree and the alignment were made using BioNumerics v7.0. (MCS, meat contact surface; NMCS, non-meat contact surface; I, insects; M, raw pork).

Total of 424 *L. monocytogenes* isolates were divided into three serotypes: 1/2c (73%), 1/2a (15%) and 3a (12%). Serotype 1/2c isolates was predominant in all sample types including raw pork, MCS, NMCS and insects (**Table 1**). All *L. monocytogenes* isolates belonged to 15 pulsotypes (PTs), and PT4 was the most prevalent pulsotype (58%), followed by PT279 (10%), PT16 (8%), PT11 (6%) and PT54 (8%) (**Figure 1**). The predominant PT4 counted for 60, 59, 68, and 45% isolates of raw pork, MCS, NMCS and insects respectively (**Table 1**). Nine sequence types (STs) were found, and the most common STs were ST9 (72%), followed by ST155 (12%), ST121 (8%) and ST8 (6%). Furthermore, ST9 was the main ST isolated from raw pork, MCS, NMCS and insects, accounting for 72, 77, 89, and 46% respectively (**Table 1**). A minimum spanning tree of the STs was constructed (**Figure 2**). Most of the STs differed by two or more alleles while ST35, ST120, and ST705 differed by one allele to ST9, ST8, and ST155 respectively (**Figure 2**). There was a good concordance for serotypes, pulsotypes and sequence types, the majority of ST9 isolates (80%) belonged to PT4 while ST155 and ST121 isolates were predominantly inclusive of PT54 (64%) and PT16 (100%) respectively (**Figure 1**). The serotypes 1/2a, 1/2c and 3a isolates were mainly belonging to ST121 (56%), ST9 (99%) and ST155 (96%) respectively.

In general, *L. monocytogenes* 1/2c/PT4/ST9 isolates were predominant (244/424, 58%), which recovered from samples of different sources (raw pork, MCS, NMCS, and insects), and accounted for 77, 70, 67, 63, 60, 59, 55, 51, 45, and 44% of *L. monocytogenes* isolates obtained from meat mincers, walls, floors, chopping board and knives, raw pork, the outer surfaces of chest freezers, hands, the inner surfaces of chest freezers, cockroaches and flies respectively. Notably, as the second predominant isolates from insects, PT16 (32%) strain presented at lower proportion of isolates from raw pork (2%), MCS (6%) and NMCS (0).

### High-Risk Booths and Repeated Contamination of *L. monocytogenes*

Based on the number of *L. monocytogenes* isolates obtained from the 65 booths, the relative risk of *L. monocytogenes* contamination among the booths was assessed. There were eight types of samples including raw pork, inner and outer surfaces of chest freezers, meat mincers, hands, floors, walls, flies and cockroaches in each booth and there were a total of 48 samples per months obtained in each booth during the 6 months. The total number of *L. monocytogenes* isolates per booth varied from one to 16 with a median of six. The majority of the booths (82%) were contaminated with one to nine *L. monocytogenes*. High-risk contamination booths were defined at the median level of six isolates or above and 37 booths (57%) were classified as high-risk booths.

**FIGURE 3:**
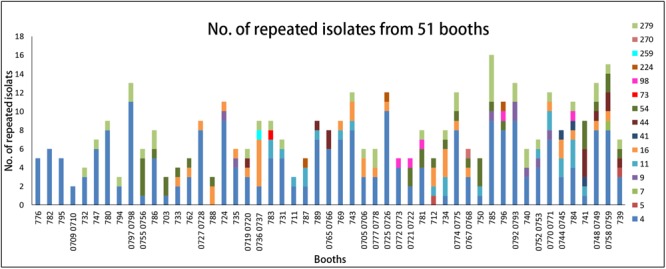
The distribution of pulsotypes of *L. monocytogenes* isolates in 51 repeated contamination booths. X-axis, booth number. Y-axis, the number of repeated PTs isolates. Legend: different colors represent different pulsotypes of *L. monocytogenes* isolates.

*Listeria monocytogenes* with the same pulsotype was repeatedly isolated from the same site (the same booth or the same sampling type) in different dates. Overall, 51 booths (51/65, 78%) were found to be contaminated repeatedly (**Figure 3**), among them only 4 booths were contaminated by a single pulsotype (PT4) strains and 40 booths were contaminated by two to four different pulsotype strains. PT4 isolates was predominant and counted for 60% of 51 repeatedly contaminated booths (**Figure 3**). Furthermore, *L. monocytogenes* isolates from different months presented similar distribution of pulsotypes, at least five pulsotypes in each month were found with PT4 predominated. The monthly isolation of *L. monocytogenes* varied from 33 to 72 and the highest prevalence appeared in May (**Supplementary Figure S1**).

**FIGURE 4:**
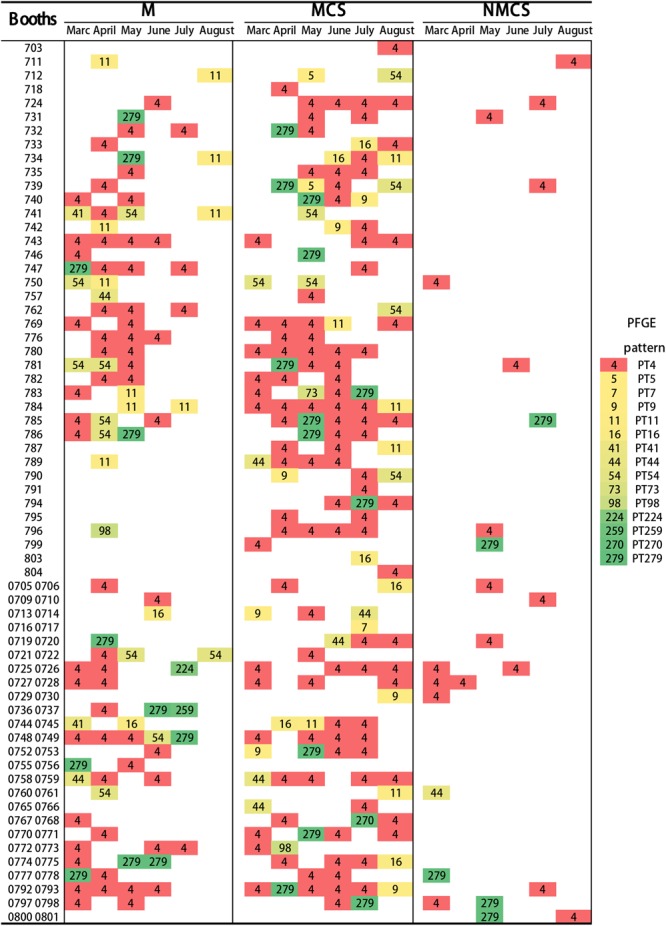
Distribution of pulsotypes of *L. monocytogenes* isolates (Raw pork, MCS and NMCS) collect from 64 booths of the retail market during six months. Every rectangle with different color and the number represents a pulsotype. (MCS, meat contact surface; NMCS, non-meat contact surface; M, raw pork).

The highest frequency of *L. monocytogenes* pulsotypes in raw pork, MCS and NMCS at each booth during the sampling period was shown in **Figure 4**. The majority of raw pork, MCS and NMCS were contaminated by *L. monocytogenes* PT4 strains. Overall, there was a higher level of contamination in raw pork and MCS samples (29% and 16%) than NMCS samples (4%) (**Figure 4**).

## Discussion

*Listeria monocytogenes* contamination in raw pork is an important food safety concern. In this study, we investigated *L. monocytogenes* contamination of raw pork, retail environment and insects in a retail market, and identified the potential risk factors.

In this study, the *L. monocytogenes* contamination rate in raw pork was 29%, which is higher than that of previous reports in China and other countries such as Chile and Japan (6–21%) (Inoue et al., 2000; Chen et al., 2015; Montero et al., 2015; Wang K. et al., 2015; Wang W. et al., 2015; Luo et al., 2017). This higher rate of *L. monocytogenes* contamination in raw pork was more likely due to the contamination in the retail market rather than upstream of the processing or retail chain, because our previous study showed that *L. monocytogenes* contamination in slaughterhouses in Beijing was very low (0.5%) (Liu et al., 2014). Our another survey showed the level of *L. monocytogenes* contamination in the pork carcass swab samples from the wholesalers which supply raw pork to the retail market was lower with 10 of the 150 (7%) tested samples being positive. and there was no *L. monocytogenes* isolated from the swab samples of the inner surfaces of meat trucks and motorized tricycles which were used for raw pork transporting in the first 3 months (unpublished data). Therefore, the main *L. monocytogenes* contamination source of raw pork possibly was not from slaughterhouse and transport links but likely from the retail market itself. MCS samples, especially chopping boards and knives were found to have a higher positive rate of *L. monocytogenes* than NMCS, which suggested that MCS would act as an important means to transmit *L. monocytogenes* to raw pork. The genotype similarity of the isolates also provided further evidence of transmission in this retail market. The predominant *L. monocytogenes* subtype strains (1/2c/PT4/ST9) isolated from environment (including MCS and NMCS) and insects were also the main subtype strains existed in raw pork. We also found that prevalence of *L. monocytogenes* was higher in months with higher temperature in our study, which was different from the patterns of report about that in southwest China (Luo et al., 2017), which possibly attributed to the difference of regional climate and retail environment.

As the report shows, most human cases of listeriosis were caused by serotype *L. monocytogenes* 1/2a, 1/2b and 4b strains (Pan et al., 2010), while serogroup 1/2a and 3a strains caused 47% of human clinical cases from 1958 to 2010 in Sweden (Lopezvalladares et al., 2014). *L. monocytogenes* strains of serotypes (1/2a, 1/2b, 1/2c), pulsotype PT4, sequence types (ST9, ST8, ST87, ST3) were the predominant foodborne strains in China (Wang et al., 2012; Luo et al., 2017), and the *L. monocytogenes* 1/2a, 1/2b, 1/2c, 3a, and 4b strains respectively belonged to ST87, ST3, ST5, ST8, and ST9 were found from the patients of listeriosis in China (Wang Y. et al., 2015). In this study, we found that 1/2c, 1/2a and 3a strains (including ST9, ST8 and PT4 as main types) existed in the raw pork. Moreover, environment and insects in the retail market were contaminated with high level by identical types *L. monocytogenes* strains, thus cross-transmission of *L. monocytogenes* among them would be an important risk factor of foodborne infection for human, considering the fact that pork is the most popular meat in China.

Insects could act as important mechanical vectors in the transmission of a variety of infectious diseases including foodborne infections (Ekdahl et al., 2005; Pava-Ripoll et al., 2015). But no knowledge about the insects as a vehicle of *L. monocytogenes* transmission was reported before in China. The *L. monocytogenes* isolation rate (20%) from flies in this study was much higher than that of reported in the United States (Pava-Ripoll et al., 2015). Considering that flies and cockroaches were abundantly presented in the retail market, they could act as a mobile vehicle for spreading *L. monocytogenes* to every surface they contacted. Thus more effective strategies for insects control should be conducted to reduce the risk of *L. monocytogenes* contamination of raw pork in the retail market.

Repeated contamination of *L. monocytogenes* in a variety of food processing environments has been reported by various studies (Nucera et al., 2010; Ferreira et al., 2011; Williams et al., 2011; Pagadala et al., 2012; Vongkamjan et al., 2013; Muhterem-Uyar et al., 2015), but the information about it in the retail market was less studied. The study by Ferreira et al. (2011) showed that PFGE typing of 240 representative *L. monocytogenes* isolates offered evidence that these strains isolates from different production dates persisted for 10–32 months. In this study, 47 of 64 booths were found to be repeatedly contaminated mainly by persistent PT4 strains, which suggested that PT4 strains were more adapted to the environment. This observation not only supported that specific *L. monocytogenes* pulsotypes could persisted over time at a retail level but also suggested that these persistent *L. monocytogenes* contributed to repeated contamination of food, which was often consumed by people. A case of *L. monocytogenes* infection reported in 1989 due to consumption of turkey franks and 30 cases of *L. monocytogenes* in 11 US states caused by delicatessen turkey in 2000 were found had a close link on contamination sources via molecular subtyping, which indicated that outbreak strains may have persisted from the same source for 12 years and caused persistent food contamination (Wenger et al., 1990; Olsen et al., 2005). Besides, some researchers have found that environmental factors have been identified as key contributors to persistence (Ferreira et al., 2014). These studies underscored environmental persistence leading to increased risk of human infections. In this study, we found the repeated contamination of *L. monocytogenes* in the environment especially in MCS (in particular the chopping boards and knives) was most serious, which would play the major role for *L. monocytogenes* transmission to the raw pork. Thus effective strategies such as cleaning and sanitizing would significantly reduce *L. monocytogenes* contamination of environment in the raw pork retail market, and then prevent the possible foodborne listeriosis in humans.

## Conclusion

This study investigated the risk factors and level of *L. monocytogenes* contamination in raw pork retail markets in China. The results showed that *L. monocytogenes* contamination was higher in raw pork and MCS than in NMCS, insects in retail market had a high carriage of *L. monocytogenes*. Cross-contamination from MCS to raw pork acted as an important risk factor to transmit *L. monocytogenes* to raw pork. Insects were an important vector for *L. monocytogenes* transmission between environment and raw pork in the retail market. Specific and effective strategies to sanitize the environment and control insects are needed to reduce raw pork contamination in retail markets and the risk of human listeriosis.

## Author Contributions

HaL performed the major experiments, statistical analysis, and wrote the manuscript. PW did lots of work in experiments. LL helped in data analysis. XC, YiW, YaW, HiL, LZ, and SJ participated in sample collection. RL and CY helped in revising the paper. CY is the corresponding author. All authors approved the final manuscript.

## Conflict of Interest Statement

The authors declare that the research was conducted in the absence of any commercial or financial relationships that could be construed as a potential conflict of interest.

## Footnotes

1 http://bigsdb.pasteur.fr/perl/bigsdb/bigsdb.pl?db=pubmlst_listeria_seqdef_public

## Supplementary Material

FIGURE S1 The distribution of pulsotypes of *L. monocytogenes* isolates from repeated booths during 6 months. X-axis: six sampling months Y-axis: the number of repeated PTs. Different colors represent different pulsotypes of *L. monocytogenes* isolates.
