# Supplementary figures and images for: Risk Factors and Level of Listeria monocytogenes Contamination of Raw Pork in Retail Markets in China
Source: Front Microbiol. 2018 May 29;9:1090. doi: 10.3389/fmicb.2018.01090 (PMC5986919; doi:10.3389/fmicb.2018.01090)

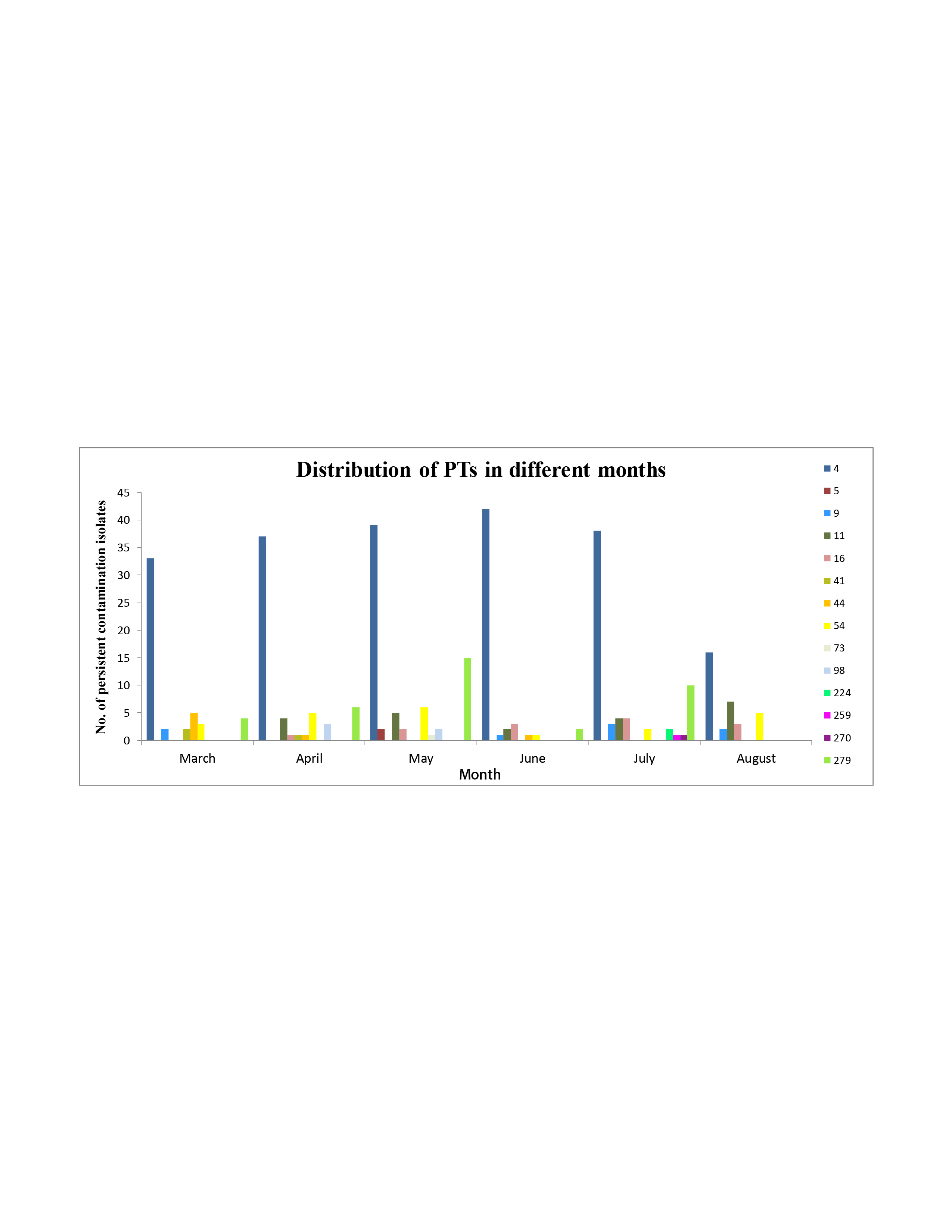

Supplement: FIGURE S1 — The distribution of pulsotypes of L. monocytogenes isolates from repeated booths during 6 months. X-axis: six sampling months Y-axis: the number of repeated PTs. Different colors represent different pulsotypes of L. monocytogenes isolates. [file Image_1.tif]
